# Procalcitonin for selecting the antibiotic regimen in outpatients with low-risk community-acquired pneumonia using a rapid point-of-care testing: A single-arm clinical trial

**DOI:** 10.1371/journal.pone.0175634

**Published:** 2017-04-20

**Authors:** Mar Masiá, Sergio Padilla, Victoria Ortiz de la Tabla, Matilde González, Cristina Bas, Félix Gutiérrez

**Affiliations:** 1 Infectious Diseases Unit, Hospital General Universitario de Elche, Universidad Miguel Hernández, Alicante, Spain; 2 Microbiology Service, Hospital Universitario de San Juan, Alicante, Spain; 3 Emergency Department, Hospital General Universitario de Elche, Elche, Spain; Ospedale Maggiore Policlinico, ITALY

## Abstract

**Objective:**

We aimed to assess the role of procalcitonin (PCT) to guide the initial selection of the antibiotic regimen for low-risk community-acquired pneumonia (CAP).

**Methods:**

A single-arm clinical trial was conducted including outpatients with CAP and Pneumonia Severity Index risk classes I-II. Antimicrobial selection was based on the results of PCT measured with a rapid point-of-care testing. According to serum PCT levels, patients were assigned to two treatment strategies: oral azithromycin if PCT was <0.5 ng/ml, or levofloxacin if levels were ≥0.5 ng/ml. Primary outcome was clinical cure rate. Short-term and long-term outcomes were assessed. Results were compared with those of a historical standard-of-care control-group treated in our centre.

**Results:**

Of 253 subjects included, 216 (85.4%) were assigned to azithromycin. Pneumococcal infection was diagnosed in 26 (12%) and 21 (56.8%) patients allocated to azithromycin and levofloxacin groups, respectively. No patients in the azithromycin group developed bacteraemia. Atypical organisms were more common in patients given azithromycin (18.5% vs 8.1%, respectively). The majority (93%) of patients with atypical pneumonia had low PCT levels. Clinical cure rates were 95.8% in the azithromycin group, 94.6% in the levofloxacin group, and 94.4% in the historical control group. No 30-day mortality or recurrences were observed, and the 3-year rates of recurrence and mortality were very low in both groups. Adverse events occurrence was also infrequent.

**Conclusion:**

A PCT-guided strategy with a rapid point-of-care testing safely allowed selecting empirical narrow-spectrum antibiotics in outpatients with CAP.

**Trial registration:**

The study is registered with ClinicalTrials.gov, number NCT02600806

## Introduction

Guidelines for empirical antimicrobial therapy for community-acquired pneumonia (CAP) aim to improve patients’ care and to reduce variability in disease management. Whereas there is a general consensus in the treatment of patients who require hospitalization, therapeutic options for outpatients with CAP vary between different guidelines. European guidelines [1–3] recommend empirical therapy with amoxicillin or penicillin. This regimen does not include coverage for atypical pathogens, which account for a significant proportion of all cases of CAP [4], and so may be associated with persistence of fever and the need for additional medical visits. By contrast, the Infectious Diseases Society of America/American Thoracic Society (IDSA/ATS) guidelines recommend a macrolide or doxycycline in patients with no comorbidity [5]. This strategy may be associated with an increased risk for therapeutic failure in areas with high prevalence of pneumococcal macrolide and doxycycline resistance [6, 7]. To avoid this, dual therapy with a macrolide plus a beta-lactam or a respiratory fluoroquinolone is recommended in those areas [5]. The last option facilitates however overuse of fluoroquinolones in many infections caused by atypical bacteria, which could be successfully treated solely with macrolides, thereby reducing fluoroquinolone side effects, including the risk for development of pneumococcal resistance [8].

Distinction between infections caused by typical and atypical respiratory pathogens is difficult and cannot be made solely on clinical grounds. The availability of biological predictors of atypical etiology might help avoiding overuse of fluoroquinolones and selecting candidates for monotherapy with macrolides in patients with CAP. Procalcitonin (PCT) is a precursor of the hormone calcitonin, and its levels have been found to increase during bacterial infection [9]. Procalcitonin has been extensively evaluated in the management of lower respiratory infections, especially to guide initiation and interruption of the antibiotic therapy [10–13]. Apart from distinguishing between bacterial and viral etiology, PCT values have also been found to be higher in CAP caused by typical, and particularly by *S*. *pneumoniae*, than by atypical bacteria like *Mycoplasma*, *Chlamydophila*, *Legionella or Coxiella* [14–18].

We postulated that PCT levels might aid differentiating the microbial atypical vs typical etiology in outpatients with CAP, and might therefore be useful to select targeted antibiotic therapy. Specifically, we used a rapid point-of-care PCT test for the initial assignment of monotherapy with macrolides vs fluoroquinolones in outpatients with CAP according to the obtained values, with the hypothesis that the strategy would be safe and would allow avoiding overuse of fluoroquinolones in an area of high prevalence of pneumococcal macrolide resistance. We compared this strategy with standard care using a historical control group of outpatients with CAP treated according to usual practice in our centre [19].

## Materials and methods

### Setting and population studied

This single-arm clinical trial was conducted at Hospital General Universitario de Elche (Alicante, Spain), a 430-bed, university-affiliated teaching hospital. Adult patients (age >15 years) seen at the emergency department (ED) who had signs and symptoms associated with pneumonia were screened for eligibility. The severity of pneumonia was calculated using the Pneumonia Severity Index (PSI) score system [20]. Inclusion criteria were fever with/without respiratory symptoms plus a new infiltrate on chest radiograph, and a PSI score ≤70 (risk classes I-II). Exclusion criteria were PSI risk classes III-V, age ≥65 years, comorbidity (diabetes, chronic obstructive pulmonary disease, chronic renal disease, neoplasm, immunosuppression including HIV infection, chronic heart failure or cirrhosis), white blood cell count ≥20.0 x 10^9^/L, pleural effusion, bilateral infiltrates, previous failure or allergy to macrolides or quinolones, and need for oxygen therapy. The study was approved by the local Ethics Committee (Ethics Committee of Hospital General Universitario de Elche) and Consellería de Sanidad, Generalitat Valenciana, Spain (DOGV 4.781), and all patients signed an informed consent. No minors/children were included. The study is registered with ClinicalTrials.gov, number NCT02600806. It was initiated before its registration because there was no randomization procedure, and the therapeutic strategy included two widely used antibiotics which constitute the standard of care for the management of CAP according to scientific guidelines recommendations. S1 Checklist shows the Transparent Reporting of Evaluations with Nonrandomized Designs (TREND) checklist of the study.

A historical control group of outpatients matched for disease and PSI risk class, and treated according to usual practice in our centre, was selected for comparison [19].

### Pneumonia clinical protocol

Based on previous data, including a study conducted in our centre, in which lower PCT levels were found among patients with low-risk CAP caused by atypical pathogens [14–18], we designed a clinical protocol to treat outpatients with azithromycin vs levofloxacin according to PCT results (S1A and S1B File). In the ED, patients meeting the above inclusion criteria had the PCT serum levels measured with a rapid test, and were assigned to any of each treatment categories as follows:

1. If PCT was <0.5 ng/ml, patients received azithromycin, 500 mg/day orally for 5 days
2. If PCT was ≥0.5 ng/ml, patients received levofloxacin, 500 mg/day orally for 7 days

The clinical protocol was implemented in May 2005 in collaboration with the hospital’s ED for the management of adult outpatients with CAP meeting the above inclusion criteria. Patients included until April 2012 have been analysed for the present study.

#### Laboratory and microbiological studies

In the ED, patients with signs and symptoms of pneumonia had a blood sample collected for routine biochemical and haematological determinations, and PCT concentration measurement.

Rapid testing for the determination of PCT was performed with BRAHMS PCT-Q, an immunochromatographic test for the semi-quantitative detection of PCT in serum (BRAHMS GmbH, 16761 Hennigsdorf, Germany). PCT concentration ranges are the following: <0.5 ng/ml; ≥ 0.5 ng/ml; ≥2 ng/ml; ≥10 ng/ml. Values below the lower limit of detection were used to assign patients to the group of “potentially atypical pathogens”. A PCT value < 0.5 ng/ml has been associated with a low likelihood of systemic infection/sepsis [21].

The etiological diagnostic workup included obtaining sputum samples from patients with productive cough, and a urine sample for detection of *S*. *pneumoniae* and *Legionella pneumophila* serogroup 1 antigens by immunochromatographic assays (BinaxNOW, Alere Healthcare SLU, Spain). Only qualified sputum samples, as defined according to standard criteria (presence of >25 WBC and <10 squamous cells per low-power magnification field [x10]) were evaluated. Serum samples (obtained during the acute stage of illness and 4 weeks later) were collected and frozen at -80°C for ulterior serological testing. An indirect chemiluminescent immunoassay (VirClia^®^ Monotest, Vircell, S.L., Granada, Spain) was performed to detect IgG antibodies against *Mycoplasma pneumoniae*, *Chlamydophila pneumoniae*, *Legionella pneumophila* and *Coxiella burnetii*. Calculation of cut-off values and interpretation of the results was performed in accordance with the instructions of the manufacturer. The diagnostic criteria were either a seroconversion (index value from negative to positive) or a significant increase in the index value (≥threefold) in paired samples. All assays were performed and analysed by the same person blinded to patient status.

#### Follow-up and outcome measures

After treatment had been assigned, patients were referred to the outpatients’ clinic, where they were seen within the following 24 hours (Visit 2). A phone visit (Visit 3) was scheduled on day 7, and the last programmed visit on day 30 at the clinic (Visit 4). Patients were instructed to visit the outpatients’ clinic if their clinical status worsened or fever persisted more than 48 hours after the first visit.

Primary outcome was clinical cure, defined as an improvement or lack of progression of baseline radiographic findings at the end of therapy (EOT) and resolution of signs, including chest X-Ray, and symptoms of pneumonia at visit 4. Failure was defined as persistence or progression of signs and symptoms or progression of radiological signs of pneumonia at EOT, persistent infiltrate on X-Ray at visit 4, and initiation within 2 calendar days of the initial antibiotic therapy of a different potentially effective antibiotic, death on or after day 3 attributable to primary infection, or relapsed infection at visit 4.

Secondary outcomes were number of participants with treatment-related adverse events, antibiotic change requirement due to toxicity, short-term (30-day) and long-term (3-year or longer) mortality, and number of 3-year recurrences, defined as new episodes of community-acquired pneumonia occurring after clinical cure of the initial episode. The long-term outcome of the patients was assessed through a structured telephone interview. Pneumonia recurrence, need for hospital admission, and death were also reviewed and checked by using the hospital electronic databases and the electronic records of the Department of Health.

### Statistical analyses

Descriptive statistics were computed by using frequency distributions, and median with interquartile ranges (IQR). For the comparisons between the study patients and those from the historical control group, we used χ2 test or Fisher’s exact test where appropriate to compare proportions, and the Mann-Whitney U-test to compare median values. Statistical analyses were performed using SPSS Version 17.0 (Chicago, IL). We estimated a sample size of 232 patients for a pre-specified threshold failure rate of 10%, with an 85% power and a two-sided alpha level of 0.05. The planned sample size was increased by 10% to 254 patients to account for losses to follow-up.

## Results

**Fig 1:**
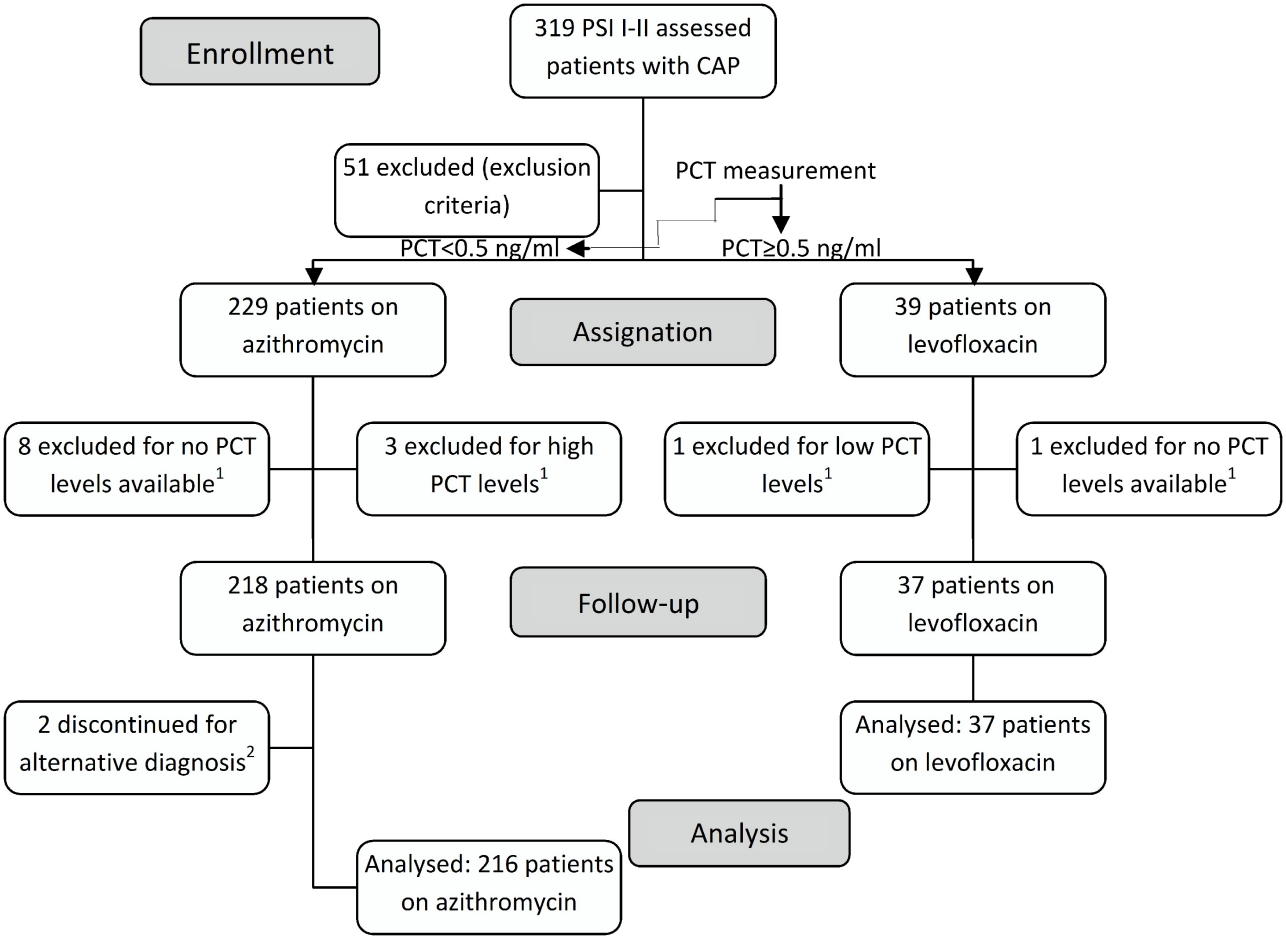
Flowchart of patients with CAP. PSI I-II indicate Pneumonia Severity Index risk classes I and II; CAP, community-acquired pneumonia; PCT, procalcitonin. ^1^Those cases were protocol violations, and were excluded from the study. ^2^One patient was diagnosed with pulmonary tuberculosis; another patient was diagnosed with pulmonary embolism.

The flowchart of the patients is shown in Fig 1. Of 319 assessed patients with PSI risk classes I-II, 253 (79.3%) patients were finally included; of them, 216 (85.4%) were assigned to azithromycin (Fig 1). No patients included in the study were lost to follow-up.

Clinical data and etiological diagnoses by treatment group are shown in Table 1.

**Table 1:** Baseline characteristics of the patients and established etiological diagnosis.

	All patients n = 253	Azithromycin group n = 216	Levofloxacin group n = 37
Sex			
Male	148 (58.5)	132 (61.1)	16 (43.2)
Female	137 (41.5)	84 (38.9)	21 (56.8)
Age, years, median (IQR)	36 (28–47.5)	36 (27.3–47)	36 (29.5–52)
PSI score, median (IQR)	34 (24–46)	34 (24–46)	35 (25–57.5)
PSI class, no. (%)			
I	189 (74.7)	166 (76.9)	23 (62.2)
II	64 (25.3)	50 (23.1)	14 (37.8)
Final etiological diagnosis			
Typical bacterial etiology	55 (21.7)	34 (15.7)	21 (56.8)
Pneumococcal pneumonia	47 (18.6)	26 (12.0)	21 (56.8)
Atypical pneumonia	43 (17.0)	40 (18.5)	3 (8.1)
Unknown etiology	160 (63.2)	145 (67.1)	15 (40.5)

Data are expressed in number (%), unless indicated

IQR, interquartile range; PSI, Pneumonia Severity Index

### Microbial aetiology and procalcitonin levels

Tables 1 and 2 show the results of the microbial studies by treatment group.

**Table 2:** Etiological agents identified by treatment group.

	All patients n = 253	Azithromycin group n = 216	Levofloxacin group n = 37
Positive urinary antigen			
Patients tested	251 (99.2)	214 (99)	37 (100)
*Streptococcus pneumoniae*	44 (17.4)	24 (11.1)	20 (54.1)
*Legionella pneumophila*	5 (1.9)	3 (1.4)	2 (5.4)
Isolation from sputum culture			
Patients	217 (85.8)	184 (85.2)	33 (89.2)
*S pneumoniae*	11 (4.3)	7 (3.2)	4 (10.8)
*Haemophilus influenzae*	6 (2.4)	6 (2.8)	0
*Klebsiella pneumoniae*	1 (0.4)	1 (0.46)	0
Other	1 (0.4)	1 (0.46)	0
Isolation from blood culture			
Patients	71 (28.1)	55 (25.5)	16 (43.2)
*S*. *pneumoniae*	2 (0.8)	0	2 (5.4)
Positive serologic results			
Patients	169 (66.8)	146 (67.6)	23 (62.2)
*Mycoplasma pneumoniae*	33 (13)	32 (14.8)	1 (2.7)
*Chlamydophila pneumoniae*	4 (1.6)	4 (1.9)	0
*L*. *pneumophila*	2 (0.8)	2 (0.9)	0
*Coxiella burnetii*	0	0	0

Data are expressed in number (%)

The etiological agent was identified in 22 (59.5%) patients placed on levofloxacin and in 71 (32.9%) patients on azithromycin (Table 1). Thirty four (15.7%) patients receiving azithromycin and 21 (56.8%) patients receiving levofloxacin were diagnosed with typical bacterial infection. In 40 (18.5%) patients included in the azithromycin and 3 (8.1%) in the levofloxacin group, respectively, an atypical microorganism was identified. A diagnosis of pneumococcal infection was established in 26 (12%) patients receiving azithromycin; of them, *S*. *pneumoniae* was isolated from the sputum culture in 7 (26.9%) patients (Table 2). In 2 of these cases, the pneumococcal isolate was resistant to macrolides, with an erythromycin MIC ≥1 μg/ml; these isolates also had intermediate susceptibility to penicillin (MIC of 0.25 and 0.12 μg/ml respectively). Pneumococcal infection was diagnosed in 21 (56.8%) patients who received levofloxacin. In 6 patients *S*. *pneumoniae* was isolated from sputum or blood cultures; one of them (16.6%), isolated from sputum, was resistant to azithromycin (MIC ≥1 μg/ml).

Procalcitonin levels were <0.5 ng/ml in 40/43 (93%) patients diagnosed with atypical pneumonia, and in 34/55 (61.8%) patients with typical bacterial pneumonia. The only 2 patients with positive blood cultures, both with pneumococcal bacteraemia, had PCT values of 10 ng/ml. No indeterminate results were obtained.

### Outcome measures

#### Treatment failure

A total of 242 (95.6%; 95% CI, 92.9%-98.4%) patients achieved clinical cure, and did not need to change their treatment regimen. In 7 (3.2%) patients receiving azithromycin, the antibiotic was changed due to treatment failure (Table 3).

**Table 3:** Short-term and long-term outcome measures.

	Azithromycin group n = 216	Levofloxacin group n = 37
Clinical cure rate	207 (95.8)	35 (94.6)
Antibiotic regimen change	9 (4.1)	2 (5.4)
Change for treatment failure	7 (3.2)	0
Change for toxicity	2 (0.92)	2 (5.4)
30-day hospital admission	2 (0.92)	0
Pneumonia recurrence^1^	5 (2.3)	0
1-year recurrence^2^	3 (1.4)	0
Time to first recurrence, months, median (IQR)	12 (11.5–25)	0
30-day mortality	0	0
3-year mortality	1 (0.5)	0
Time to death, months	23.8	-
Cause of death		
Neoplastic disease^3^	1	-

Data are expressed in number (%), unless indicated

IQR, interquartile range

^1^Number of recurrences within 3 years after diagnosis

^2^Number of recurrences within the first year after diagnosis

^3^Lung cancer

In 4 of them, the reason for change was pleural effusion development, with no microorganisms recovered from pleural fluid, and a positive pneumococcal urinary antigen in 1 patient. Of the remaining 3 patients failing to respond to azithromycin, the urinary pneumococcal antigen test was positive in 1, and in 2 patients, no microbial diagnosis was achieved; none of them needed hospital admission. No patient with macrolide resistant isolates experienced clinical failure. None of the 37 patients included in the levofloxacin group had to change the antibiotic for treatment failure.

#### Adverse event development leading to drug discontinuation

Adverse events occurrence was infrequent. Two patients initially receiving azithromycin were changed to levofloxacin because of rash and diarrhea, respectively. Two patients on levofloxacin were also changed due to adverse events; one patient was changed to azithromycin due to skin rash development; another patient was changed to intravenous levofloxacin because of vomiting. There were no differences between groups in the rate of 30-day hospital admissions.

#### Pneumonia recurrence

Patients were followed-up for 36 months after the diagnosis of CAP. Pneumonia recurrence was only observed in 2.3% patients receiving azithromycin (Table 3). All recurrences occurred more than 90 days after the diagnosis of pneumonia. No recurrences were observed in the two patients with pneumococcal macrolide-resistant isolates who received azithromycin.

#### Mortality

No patient died within the first 30 days after CAP diagnosis in either group. Long-term mortality was very low, and not different between groups (Table 3).

#### Historical control group

Outpatients included in a prospective cohort study of 493 adult patients with community-acquired pneumonia carried out in our centre from 1999 to 2001 were selected for comparison [17]. The PSI score [18] had also been used in that cohort to classify patients according to severity. For comparability with our study group, we selected only patients with PSI risk classes I and II who met the inclusion/exclusion criteria. The sample comprised 124 patients, of them 63 (50.8%) were men (P = 0.91 compared to patients of the clinical pathway). Characteristics of the patients and comparison to the study group are provided in Table 4. Patients from this group were slightly younger (median [IQR] age 34 [25.3–50.8] years vs 36 [28–47.5]; P<0.001), and had a somewhat lower PSI score (31 [20.3–45.5] vs 34 [24–46]; P<0.001). Microbial diagnosis was achieved in 70 (56.4%) patients; 26 (21%) patients were diagnosed with typical bacterial pneumonia, of them 21 (16.9%) with pneumococcal pneumonia; 35 (28.2%) patients were diagnosed with atypical bacterial pneumonia, and 19 (15.3%) of them seroconverted for *Mycoplasma pneumoniae*; 6 (4.8%) patients with viral pneumonia; and 3 (2.4%) patients with mixed (bacterial plus atypical) pneumonia. Antibiotic therapy consisted of fluoroquinolones in 39 (31.5%) patients (P = 0.0002 compared with patients of the PCT-guided strategy), macrolides in 30 (24.2%), beta-lactam antibiotics in 23 (18.5%), and combined therapy in 29 (23.4%). Clinical cure was achieved in 117 (94.4%; 95% CI, 89.3%-98.8%) patients (P = 0.44 compared to the clinical pathway intervention); antibiotic regimen was changed for pleural effusion development in 5 patients, all of them subsequently admitted to hospital, and in 2 patients for absence of clinical response. No patient died during the 30-day period.

**Table 4:** Comparison between the historical control and the study group.

	Historical control group n = 124	Study group n = 253	P^1^
Sex, male	63 (50.8)	148 (58.5)	0.91
Age, years, median (IQR)	34 (25.3–50.8)	36 (28–47.5)	<0.001
PSI score, median (IQR)	31 (20.3–45.5)	34 (24–46)	<0.001
Final etiological diagnosis	70 (56.4)	93 (36.8)	0.0004
Typical bacterial etiology	26 (21)	55 (21.7)	0.89
Pneumococcal pneumonia	21 (16.9)	47 (18.6)	0.77
Atypical pneumonia	35 (28.2)	43 (17.0)	0.014
Unknown etiology	54 (49.2)	160 (63.2)	0.0004
Clinical cure	117 (94.4)	242 (95.6)	0.44

Categorical data are expressed in number (%), unless indicated.

IQR, interquartile range; PSI, pneumonia severity index

^1^χ2 test or Fisher’s exact test were used to compare proportions, and the Man-Whitney test to compare median values

## Discussion

Our study demonstrates the short-term and long-term safety and efficacy of an intervention guided by PCT levels for the election of the antibiotic regimen in outpatients with CAP. When it was implemented to use azithromycin monotherapy in an area of high prevalence of pneumococcal macrolide resistance, a high clinical cure rate was achieved, that was comparable to the rate of responses observed with the standard strategy of the historical control group. By contrast, compared with usual care, our strategy allowed narrowing the antibacterial spectrum and avoiding overuse of fluoroquinolones.

To the best of our knowledge, this is the first study to use a PCT-guided intervention to select the antibiotic regimen composition in CAP. Until now, the aim of PCT-based strategies was to safely reduce the antibiotic consumption [14–18], but the ability of PCT to discriminate between classic and atypical bacteria had not been exploited to guide the initial empirical antibiotic choice in patients with CAP. In contrast to other studies, we used a rapid point-of-care testing to measure the PCT, which allows an immediate categorization of the patients at the Emergency Department or at Primary Care. The strategy was accompanied by a low rate of clinical failure, which was not different from that found with the standard treatment approach, suggesting that the PCT levels classified properly the majority of patients. No early recurrences occurred, and the long-term follow-up showed a very low rate of new pneumonia episodes.

*S*. *pneumoniae* still constitutes the most frequent aetiology of CAP [19, 22–24]. Resistance to macrolides is currently a concern worldwide, with a rising frequency described in the United States [6], and a high prevalence in Southern and Eastern Europe and Asia, including Spain [7, 25, 26]. Although the clinical relevance of macrolide resistance had been questioned, observational data support its association with treatment failure [27, 28]. Our strategy allowed narrowing considerably the range of candidates to receive the broad-spectrum pneumococcal-directed antibiotic therapy recommended by the IDSA/ATS guidelines. Of note, there was a significantly higher proportion of typical bacterial infections and lower frequency of atypical bacteria in patients included in the levofloxacin-therapy group than in those who received azithromycin. Moreover, the vast majority of patients in the levofloxacin group in whom the aetiology could be identified were diagnosed with typical bacterial infection.

A majority of outpatients with CAP in our study were candidates to receive monotherapy with azithromycin. Atypical pathogens represent the second most frequent organisms causing CAP after *S*. *pneumoniae*, especially in outpatients [29–32], as we have corroborated in our study. In the group of patients with low PCT levels, the proportion of atypical organisms was even higher than that of typical bacteria. The frequency of atypical organisms was probably still greater within this subgroup, since serologic studies were only performed in two thirds of the patients, and viral pathogens were not assessed in our study. Treatment of this subgroup with a macrolide instead of amoxicillin carries a significant lower risk of treatment failure. This may generate less health care reutilization for persistence of symptoms, may be associated with an earlier clinical recovery and lower duration of the incapacity, and may consequently save costs.

We observed that the vast majority of patients with atypical pathogens had low PCT levels. Although PCT was also low in half of patients with typical bacterial infection, monotherapy with azithromycin was also successful in those cases, including the patients with macrolide-resistant pneumococcal pneumonia. By contrast, the 2 patients with positive blood cultures in the levofloxacin group had very high PCT values, and no patient in the azithromycin group developed bacteraemia. Because PCT is also a predictor of the clinical outcome [17], the low PCT levels found in patients with typical bacterial pneumonia might reflect the selection for the subgroup of patients with a higher probability of favourable outcome and, consequently, of treatment success independently of the microbial aetiology.

A limitation of the study is that the semi-quantitative method used for PCT determination is less sensitive than the quantitative PCT. However, the rapid immunochromatographic testing used in our study has the advantage of being easily performed by non-trained personnel, results are available in only 20 minutes, and therefore it does not prolong the time in ER or in the physician’s office, and it has an affordable cost. Primary care doctors and emergency physicians are thus enabled to make a rapid point-of-care decision about the empiric antibiotic therapy to be used. For safety reasons, we excluded from the PCT-guided strategy to patients with comorbidity, older age, or certain clinical findings potentially associated with a poorer outcome [33]; this strategy is therefore primarily applicable to younger patient populations with no comorbidities. However, a very low proportion of patients with PSI risk scores I-II actually had to be excluded for this reason, with a majority of outpatients with CAP being candidates to be managed with the PCT-guided intervention. The X-ray pulmonary infiltrates may persist for longer than 4 weeks, and this criterion might have introduced false positive failures. There were minor discrepancies in baseline characteristics between the historical control and the study group, although those differences favoured the control group in terms of a higher probability of clinical response. This is a single centre study, and resistance patterns as well as antibiotic prescribing practices differ across institutions and regions. Finally, false PCT positive and negative values cannot be excluded. Although the study was limited by the high frequency of cases with unknown aetiology, we were able to relate the PCT values with the microbial diagnoses obtained from the study, to verify that most atypical bacteria were associated with low PCT levels. Our results are similar to those recently obtained by Branche et al. when measuring serum PCT levels in patients with nonpneumonic viral respiratory infections [18]. In addition, the long-term follow-up data of the patients is further evidence for the efficacy and safety of the intervention. Our results should encourage the design of a clinical trial to definitively establish the role of PCT in selecting antimicrobial therapy in outpatients with CAP.

In conclusion, a strategy guided by PCT levels to treat outpatients with CAP using a rapid point-of-care testing allowed narrowing the antibacterial spectrum in a high proportion of patients. The intervention was accompanied by a high rate of clinical response, comparable to that obtained with usual practice, and by no short-term and rare long-term recurrences. Implementation of this strategy might help clinicians to select the most appropriate initial empirical antibiotic regimen, and may be advantageous in terms of costs and ecological impact.

## Supporting information

S1 Checklist TREND checklist. (PDF)

S1 File Original clinical pathway for the management of outpatients with CAP. (PDF)

S2 File Translated version of the clinical pathway for the management of outpatients with CAP. (PDF)
