# Supplementary material for: Procalcitonin for selecting the antibiotic regimen in outpatients with low-risk community-acquired pneumonia using a rapid point-of-care testing: A single-arm clinical trial
Source: PLoS One. 2017 Apr 20;12(4):e0175634. doi: 10.1371/journal.pone.0175634 (PMC5398537; doi:10.1371/journal.pone.0175634)
Supplement: S1 File — (PDF) [file pone.0175634.s002.pdf]

TÍTULO: Vía clínica basada en los niveles de procalcitonina para la atención de la neumonía adquirida en la comunidad que no precisa ingreso hospitalario

PALABRAS CLAVE: Vía clínica, neumonía adquirida en la comunidad, tratamiento ambulatorio, evaluación, indicadores.

INVESTIGADOR PRINCIPAL: Mar Masiá Canuto

INSTITUCIÓN: Hospital General Universitario de Elche

DIRECCIÓN: Camí de la Almazara, 11

C. POSTAL: 03203

LOCALIDAD: Elche

PROVINCIA: Alicante

TELÉFONO: 96 6679154

1. RESUMEN :

Objetivo: (1) Sistematizar y ordenar el proceso asistencial de la neumonía adquirida en la comunidad que no precisa ingreso hospitalario a través de la elaboración de una vía clínica y definir los indicadores para su evaluación. (2) Evaluar el papel de la procalcitonina medida con un test rápido para seleccionar la composición del tratamiento antibiótico en pacientes con neumonía comunitaria que no precisa ingreso. (3) Evaluar la eficacia y seguridad de la intervención y compararlas con un grupo de control histórico (4) Determinar la mortalidad a corto (30 días) y largo plazo (al menos 3 años) y la frecuencia de recurrencias.

Diseño: Estudio prospectivo de base poblacional.

Ámbito del estudio: Área sanitaria del Baix Vinalopó.

Sujetos de estudio: Pacientes con neumonía adquirida en la comunidad que no precisan ingreso hospitalario (estadios I y II de Fine).

Instrumentalización: Se realizará a todos los pacientes anamnesis, exploración física, RX de tórax, recogida de muestras para analítica que incluirá procalcitoninar y estudios microbiológicos y dispensación e instrucciones sobre el tratamiento antibiótico que deben seguir.

Determinaciones: Identificación de los principales patógenos implicados en la neumonía a través del cultivo de esputo y determinación de antígenos en orina. Investigación de diversas variables como respuesta al tratamiento, incidencia de complicaciones, efectos adversos, recurrencias, mortalidad, cumplimiento de la vía clínica y estimación del impacto sobre la efectividad y la eficiencia.

2. ANTECEDENTES Y JUSTIFICACIÓN DEL ESTUDIO:

La variabilidad en la práctica clínica o disparidad de criterios para atender un mismo proceso médico es un hecho frecuente que supone una amenaza sobre la efectividad de los servicios sanitarios prestados, la eficiencia de su gestión y la equidad en su provisión (1). Las vías clínicas son instrumentos que permiten planificar y coordinar la atención sanitaria de un grupo de pacientes con una patología bien delimitada y un curso clínico previsible, a través de la definición del conjunto de actividades e intervenciones asistenciales a realizar por los médicos, enfermeras y otros profesionales sanitarios, ordenándolas de forma secuencial en el tiempo (2). Con ello pretenden contribuir a hacer un uso más adecuado, racional y coordinado de los recursos sanitarios existentes para conseguir la máxima eficiencia y calidad en el proceso asistencial.

La neumonía adquirida en la comunidad (NAC) es un proceso grave y frecuente. En el área sanitaria del Baix Vinalopó la incidencia es de 12 casos por 10.000 habitantes por año, lo que supone una causa importante de morbilidad, mortalidad y una considerable carga económica. La evidencia disponible indica que existe una variabilidad significativa en el manejo clínico de la NAC (3, 4) y que existen diversos aspectos en la atención de esta patología que son subsidiarios de ser mejorados (5, 6). La aplicación de una vía clínica a los pacientes con NAC podría ayudar a optimizar el manejo de esta patología. De hecho, se han descrito diferentes intervenciones encaminadas a mejorar la atención a estos pacientes que contribuyen también a mejorar su evolución clínica, como la valoración de

la severidad de la neumonía en el momento del diagnóstico, el tiempo transcurrido hasta recibir la primera dosis del antibiótico o la prescripción apropiada del antibiótico (7).

En los últimos años se ha evaluado el impacto de la aplicación de una vía clínica a los pacientes con NAC, fundamentalmente los que precisan ingreso hospitalario y se ha encontrado mejoría en la calidad de atención a los pacientes (8-10). Sin embargo, existe escasa experiencia sobre la utilidad de la implantación de una vía clínica en los pacientes con NAC que no precisan ingreso en el hospital. Desde un punto de vista teórico, la sistematización del proceso asistencial de la NAC que no precisa ingreso hospitalario a través de la introducción de una vía clínica permitiría un ahorro de ingresos hospitalarios a través de la identificación de pacientes de bajo riesgo según el índice de severidad de PORT (11), un ahorro de pruebas diagnósticas innecesarias, una mejoría en la evolución clínica de los pacientes con la introducción de medidas como tratamiento antibiótico precoz o tratamiento parenteral inicial en casos seleccionados y un aumento de la eficiencia en la atención de esta patología (12).

La procalcitonina (PCT) es un precursor de la hormona calcitonina y sus niveles se han encontrado elevados durante la infección bacteriana [13]. En pacientes con NAC, los niveles PCT se han relacionado con la etiología microbiana; la NAC bacteriana típica, particularmente la infección neumocócica, se ha asociado a niveles significativamente más elevados de PCT que la infección por patógenos atípicos (*Mycoplasma, Chlamydophila, Legionella, Coxiella*) o neumonía viral [14-18]. La PCT ha sido evaluada extensamente en el

tratamiento de infecciones respiratorias bajas, especialmente para guiar el inicio e interrupción del tratamiento antibiótico [18-21].

3. BIBLIOGRAFÍA

1. Esteve M, Marbá L, Zaldívar C, Verdaguer A, Serra-Prat M, Berenguer J. Evaluación de la puesta en marcha de 54 trayectorias clínicas en el Hospital de Mataró. Rev Calidad asistencial 2001; 16: 722-28.
2. Carrasco G, Ferrer J. Las vías clínicas basadas en la evidenciaz como estrategia para la mejora de la calidad: metodología, ventajas y limitaciones. RTVE calidad Asistencial 2001; 16: 199-207.
3. Laurichesse H, Robin F, Gerbaud L, Pochet P, Gourdon F, Beytout J, Rey M. Empirical therapy for nonhospitalized patients with community-acquired pneumonia. Study Group of General Practitioners. Eur Respir J 1998; 11 :73-8.
4. Woodhead M, Gialdroni Grassi G, Huchon GJ, Leophonte P, Manresa F, Schaberg T. Use of investigations in lower respiratory tract infection in the community: a European survey. Eur Respir J 1996; 9: 1596-600.
5. Minogue MF, Coley CM, Fine MJ, Marrie TJ, Kapoor WN, Singer DE. Patients hospitalized after initial outpatient treatment for community-acquired pneumonia. Ann Emerg Med 1998; 31: 376-80.
6. Metlay JP, Kapoor WN, Fine MJ. Does this patient have community-acquired pneumonia? Diagnosing pneumonia by history and physical examination. JAMA 1997; 278: 1440-5.
7. Nathwani D, Williams F, Winter J, Winter J, Ogston S, Davey P. Use of indicators to evaluate the quality of community-acquired pneumonia management. Clin Infect Dis 2002; 34: 318-23
8. Meehan TP, Weingarten SR, Holmboe ES, Mathur D, Wang Y, Petrillo MK, Tu GS, Fine JM. A statewide initiative to improve the care of hospitalized pneumonia patients: The Connecticut Pneumonia Pathway Project. Am J Med 2001; 111: 203-10.
9. Marrie TJ, Lau CY, Wheeler SL, Wong CJ, Vandervoort MK, Feagan BG. A controlled trial of a critical pathway for treatment of community-acquired pneumonia. CAPITAL Study Investigators. Community-Acquired Pneumonia Intervention Trial Assessing Levofloxacin. JAMA 2000; 283: 749-55.
10. Benenson R, Magalski A, Cavanaugh S, Williams E. Effects of a pneumonia clinical pathway on time to antibiotic treatment, length of stay, and mortality. Acad Emerg Med 1999; 6: 1243-8.

11. Fine MJ, Auble TE, Yealy DM, Hanusa BH, Weissfeld LA, Singer DE, Coley CM, Marrie TJ, Kapoor WN. A prediction rule to identify low-risk patients with community-acquired pneumonia. N Engl J Med 1997; 336:243-50.
12. Nathwani D, Rubinstein E, Barlow G, Davey P. Do guidelines for community-acquired pneumonia improve the cost-effectiveness of hospital care? Clin Infect Dis 2001; 32: 728-41.
13. Becker KL, Nylen ES, White JC, Muller B, Snider RH Jr. Procalcitonin and the calcitonin gene family of peptides in inflammation, infection, and sepsis: a journey from calcitonin back to its precursors. J Clin Endocrinol Metab. 2004;89:1512–25
14. Krüger S, Ewig S, Papassotiriou J, Kunde J, Marre R, von Baum H, et al. Inflammatory parameters predict etiologic patterns but do not allow for individual prediction of etiology in patients with CAP – Results from the German competence network CAPNETZ. *Respir Res. 2009;* 10: 65.
15. Müller F, Christ-Crain M, Bregenzer T, Krause M, Zimmerli W, Mueller B, et al. Procalcitonin levels predict bacteremia in patients with community-acquired pneumonia: a prospective cohort trial. *Chest.* 2010;138:121-9.
16. Prat C, Domínguez J, Andreo F, Blanco S, Pallarés A, Cuchillo F, et al. Procalcitonin and neopterin correlation with aetiology and severity of pneumonia. *J Infect.* 2006;52:169-77
17. Masiá M, Gutiérrez F, Shum C, Padilla S, Navarro JC, Flores E, et al. Usefulness of procalcitonin levels in community-acquired pneumonia according to the patients outcome research team pneumonia severity index. Chest. 2005; 128:2223-9
18. Branche AR, Walsh EE, Vargas R, Hulbert B, Formica MA, Baran A, et al. Serum Procalcitonin Measurement and Viral Testing to Guide Antibiotic Use for Respiratory Infections in Hospitalized Adults: A Randomized Controlled Trial. J Infect Dis. 2015 [Epub ahead of print]
19. Christ-Crain M, Jaccard-Stolz D, Bingisser R, Gencay MM, Huber PR, Tamm M, et al. Effect of procalcitonin-guided treatment on antibiotic use and outcome in lower respiratory tract infections: cluster-randomised, single-blinded intervention trial. Lancet. 2004;363:600-7.
20. Kristoffersen KB, Søgaard OS, Wejse C, Black FT, Greve T, Tarp B, et al. Antibiotic treatment interruption of suspected lower respiratory tract infections based on a single procalcitonin measurement at hospital admission--a randomized trial. Clin Microbiol Infect. 2009;15:481-7
21. Schuetz P, Müller B, Christ-Crain M, Stolz D, Tamm M, Bouadma L, et al. Procalcitonin to initiate or discontinue antibiotics in acute respiratory tract infections. Cochrane Database Syst Rev. 2012;9:CD007498

4. HIPÓTESIS Y OBJETIVOS CONCRETOS

Hipótesis:
La sistematización del proceso asistencial de la neumonía adquirida en la comunidad que no precisa ingreso hospitalario a través de la elaboración y aplicación de una vía clínica permitiría mejorar la efectividad y la eficiencia en la atención de esta patología. El uso de la procalcitonina para guiar la elección del tratamiento antibiótico es una estrategia segura que permitirá ahorrar el uso de fluorquinolonas

Objetivos concretos:

1. Sistematizar y ordenar el proceso asistencial de la neumonía adquirida en la comunidad que no precisa ingreso hospitalario a través de la elaboración de una vía clínica y definir los indicadores para su evaluación.
2. Evaluar el papel de la procalcitonina medida con un test rápido para seleccionar la composición del tratamiento antibiótico en pacientes con neumonía comunitaria que no precisa ingreso hospitalario.
3. Evaluar la eficacia y seguridad de la intervención y comparalos con un grupo de control histórico
4. Determinar la mortalidad a corto (30 días) y largo plazo (al menos 3 años) y la frecuencia de recurrencias.

Criterios de valoración de respuesta

<u>Criterio primario de valoración</u>:

- Curación clínica: (mejoría clínica o ausencia de progresión de los hallazgos radiológicos basales al final del tratamiento y resolución de los signos, incluyendo la RX de tórax, y os síntomas de neumonía)

<u>Criterios secundarios de valoración</u>:

- Número de participantes con efectos adversos asociados al tratamiento
- Mortalidad (en olos sisguientes 30 días y durante los 3 años siguientes o después
- Recurrencias (después de la resolución del episodio inicial hasta el final del seguimiento, al menos durante los siguientes 3 años)

5. METODOLOGÍA.

Sujetos del estudio: Todos los pacientes atendidos en el hospital por neumonía adquirida en la comunidad que no precisen ingreso hospitalario, es decir, que pertenezcan a los estadios I y II de Fine.

Diseño: Estudio prospectivo de base poblacional.

Variables: Para evaluar la vía clínica:

Recogida y análisis de datos: - Se diseñarán unas hojas para recogida de datos en las que se incluirán todas las visitas realizadas por los pacientes al hospital. Se recogerán datos referentes a la identificación de los pacientes, características clínicas, procedimientos realizados, tratamiento suministrado y tiempo transcurrido desde la llegada al hospital y administración de la primera dosis y desarrollo de complicaciones.

- Análisis de los datos: se determinarán los indicadores de la vía clínica través del cálculo de las proporciones previamente descritas.

Dificultades y limitaciones del estudio:

- Coordinación adecuada entre los Servicios de Urgencias y la Unidad de Infecciosas para asegurar buena adherencia al protocolo.
- Reclutamiento insuficiente de pacientes en el período de 3 meses del estudio piloto que permita hacer los ajustes adecuados para la implantación de la vía definitiva.
- Dificultad en el cálculo de los indicadores de aumento de eficiencia por la necesidad de utilizar datos retrospectivos para la comparación de la atención pre y postaplicación de la vía clínica.

6. DURACIÓN Y FASES PREVISTAS:

1. Elaboración de la vía clínica y de los indicadores de evaluación
2. Estudio piloto de 3 meses de duración
3. Elaboración de la vía clínica definitiva
4. Implantación de la vía clínica en el hospital
5. Reevaluación trimestral
6. Valoración de los indicadores de impacto transcurrido 1 año desde el comienzo del estudio piloto

CRITERIOS DE INCLUSIÓN: Los pacientes deben cumplir los 3 siguientes criterios:

- ❑ Presentación aguda de fiebre y/o alguno de los siguientes signos/síntomas: cambio en el patrón de tos con/sin expectoración, dolor torácico pleurítico, disnea, auscultación pulmonar patológica

  +

- ❑ Opacidad en RX de tórax compatible con la presencia de neumonía

  +

- ❑ Fine ≤ 70 puntos

CRITERIOS DE EXCLUSIÓN:

1. Edad < 65 años
2. Comorbilidad
3. Derrame pleural
4. Afectación bilateral
5. Leucocitosis > 20.000
6. Factores de riesgo de aspiración
7. Fracaso previo o alergia a macrolidos or quinolonas
8. Necesidad de oxigenoterapia

CALENDARIO DE VISITAS:

- Se realizarán un total de 4 evaluaciones (Visitas 1, 2, 3 y 4), una de ellas (Visita 3) por contacto telefónico.

En el Anexo III se detalla el Calendario de Visitas de los pacientes.

PROCEDIMIENTOS:

1. Anamnesis y exploración física: Visita 1
2. Toma de constantes vitales: Visitas 1 y 2.
3. Recogida de esputo para cultivo: Visita 2
4. Recogida de orina para determinación de antígenos: Visita 2
5. Extracción de sangre para analítica: Visitas 1 y 2

6. Extracción de suero para congelar: Visitas 2 y 4
7. Realización de RX de tórax: Visitas 1 y 4.
8. Sólo cuando se cumplan las condiciones que se detallan, extracción de hemocultivos o de gasometría arterial: Vistas 1 ó 2
9. Administración de tratamiento oral de la primera/s dosis antibiótico y elaboración de pauta hasta su finalización, incluida cumplimentación de receta médica: Visitas 1 a 2.
10. Elaboración de informe de alta: Visita 4.

INDICADORES PARA LA EVALUACIÓN DE LA VÍA CLÍNICA:

a) Indicadores del grado de cumplimiento de la vía clínica:

1. Número de enfermos que finalizan la vía clínica x 100/ Número de enfermos que son atendidos por neumonía que no precisa ingreso. Estándar: >90%.

2. Tiempo medio transcurrido desde la llegada al hospital hasta la administración de la primera dosis del antibiótico. Estándar: < 4 horas

b) Indicador de efectos adversos:

Nº de enfermos con NAC sin ingreso que presentan algún efecto adverso (necesidad de ingreso hospitalario por empeoramiento clínico, toxicidad por los antibióticos, desarrollo de empiema...) x 100/ Nº de enfermos con NAC sin ingreso que siguen la vía clínica. Estándar: ≤ 5%.

Nº de enfermos que fallece/número pacientes atendidos en la vía clínica

c) Indicador de satisfacción:

Nº de enfermos con NAC sin ingreso que siguen la vía y contestan con 8 ó más en la pregunta de la atención recibida en la encuesta x 100/ Nº de enfermos con NAC sin ingreso que siguen la vía y contestan a la encuesta de satisfacción. Estándar: ≥ 98%.

d) Indicador de evaluación económica:

Nº de enfermos con NAC sin ingreso y siguen la vía con coste adecuado x 100/ Nº de enfermos con NAC sin ingreso que siguen la vía. Estándar: > 90%.

INDICADORES DEL IMPACTO DE LA VÍA CLÍNICA:

a) Efectividad:

- Proporción de pacientes que se cura sin necesidad de desvíos del protocolo de la vía clínica
- Disminución en el uso de antibioterapia de amplio espectro
- Tiempo medio transcurrido desde la llegada al hospital hasta la administración de la primera dosis del antibiótico. Estándar: < 4 horas
- Proporción de pacientes que no precisa cambio del tratamiento prescrito inicialmente

b) Eficiencia:

- Ahorro en el consumo de antibióticos de amplio espectro
- Ahorro en la necesidad de visitas al hospital.

DESCRIPCIÓN DEL PROCESO ASISTENCIAL:

VISITA 1 (Día 1)
Ámbito: Servicio de Urgencias o Unidad de Enfermedades Infecciosas

b) Criterios de inclusión:

- ❑ Presentación aguda de al menos 1 de los siguientes signos/síntomas: fiebre, cambio en el patrón de tos con/sin expectoración, dolor torácico pleurítico, disnea, auscultación pulmonar patológica

+

- ❑ Opacidad en RX de tórax compatible con la presencia de neumonía
- ❑ Fine ≤ 70 puntos

b) Evaluación médica

1. Anamnesis.
2. Exploración física.

c) Determinaciones y tests realizados por enfermería:

1. Constantes vitales: medida de la tensión arterial y de la temperatura
2. Extracción de sangre para analítica urgente (bioquímica y hemograma)
3. Extracción de gasometría arterial en los siguientes casos: taquipnea, disnea, hipotensión arterial, mal estado general del paciente.
4. Extracción de 2 hemocultivos en los siguientes casos: hipotensión arterial, tiritona en el momento de la evaluación, hipotermia<35ºC ó hipertermia>=40ºC.

d) Criterios para manejo ambulatorio:

Puntuación según la escala de Fine (ver Anexo 1, al final) menor ó igual a 70 puntos, o estadíos I y II.

e) Instauración de tratamiento:

- ❑ Procalcitonina < 0.50 ug/L: Azitromicina, 500 mg/día VO

- ❑ Procalcitonina ≥ 0.50 ug/L : Levofloxacino, 500 mg/día V.O

f) Citación para reevaluación:

Remitir al paciente a la Unidad de Enfermedades Infecciosas para ser visto al día siguiente (*Visita 2*).

VISITA 2 (Día 2)
Ámbito: Unidad de Enfermedades infecciosas

a) Reevaluación médica

b) Determinaciones y tests realizados por enfermería:

1. Medida de la temperatura y, sólo en los siguientes casos, de la tensión arterial: hipotensión arterial el día previo, tiritona en el momento de la evaluación, signos de hipoperfusión (sudoración importante, frialdad), mal estado general.
2. Extracción de sangre para analítica general (bioquímica completa y hemograma)
3. Extracción de suero archivo para congelar
4. Recogida de orina para determinación de antígenos de neumococo y *Legionella*
5. Recogida de esputo para Gram y cultivo
6. Extracción de gasometría arterial en los siguientes casos: taquipnea, disnea, hipotensión arterial, mal estado general del paciente.
7. Extracción de 2 hemocultivos en los siguientes casos: hipotensión arterial, tiritona en el momento de la evaluación, hipotermia<35ºC ó hipertermia>=40ºC.

c) Instauración de tratamiento:

- ❑ Procalcitonina **<** 0.50 ug/L: Azitromicina, 500 mg/día VO 5 días
- ❑ Procalcitonina ≥ 0.50 ug/L : Levofloxacino, 500 mg/día V.O 10 días

d) Citación para reevaluación:

Citar para *Visita 3*, que se realizará por contacto telefónico, en el plazo de 1 semana, o antes si se considera indicado por el estado clínico del paciente.

VISITA 3 (día 7): <u>CONTACTO TELEFÓNICO</u>

a) Evaluación médica

1. Valoración de evolución clínica y respuesta al tratamiento
2. Valoración de los resultados de los tests realizados en la visita 2

b) Citación para reevaluación:

Cita para *Visita 4* en un plazo de 3 semanas. Se solicitará RX de tórax para que esté disponible en la Visita 4 y analítica (suero archivo para congelación y analítica general si indicado).

VISITA 4 (día 30)
Ámbito: Unidad de Enfermedades Infecciosas

Evaluación médica:

1. Valoración de evolución clínica y respuesta al tratamiento
2. Valoración de la RX de tórax de control

Determinaciones y tests realizados por enfermería:

1. Extracción de suero de archivo para congelación.
2. Se extraerá también una analítica general sólo en los casos en los que existan hallazgos patológicos significativos en la analítica previa.
3. Alta de la Unidad de Infecciosas, excepto si extracción de analítica general. En esta caso, se le informará del resultado al paciente telefónicamente en los días siguientes.
4. Elaboración de informe de alta.

ANEXO 1. ESCALA DE FINE

CARACTERÍSTICA	PUNTOS
**Edad**	
Hombres	Edad en años
Mujeres	Edad en años menos 10
**Residente en hogares geriátricos, de rehabilitación**	10
**Enfermedad coexistente**	
Enfermedad neoplásica[1]	30
Enfermedad hepática[2]	20
Falla cardiaca congestiva[3]	10
Enfermedad cerebro vascular	10
Enfermedad renal	
**Hallazgos del examen físico**	
Estado mental alterado[4]	20
Frecuencia respiratoria ≥ 30/minuto	20
Presión sistólica sanguínea < 90 mm Hg	20
Temperatura < 35 °C ó ≥ 40 °C	15
Pulso ≥ 125 latidos por minuto	10
**Hallazgos de laboratorio y radiográficos**	
pH arterial < 7.35	30
Nitrógeno ureico sanguineo ≥ 30 mg/dL	20
Sodio serico < 130 mmol/L	20
Glucosa serica ≥ 250 mg/dL	10
Hematocrito < 30%	10
Presión parcial de oxigeno < 60 mm Hg*	10
Derrame pleural	10

[1]Neoplasia: cualquier cáncer excepto el basal o escamoso de piel, activo en el momento del diagnostico de la NAC o diagnosticado en el ano previo.
[2]Enfermedad hepatica: cirrosis u otras hepatopatías crónicas o hepatitis crónica activa
[3]Insuficiencia cardíaca congestiva: disfunción ventricular documentada por hallazgos clínicos, radiológicos, ecocardiográficos o ventriculografía.
[4]Alteración del estado mental. Desorientación en el tiempo, personal o en el espacio, que no sea crónico

Cálculo de riesgo de Fine: la puntuación total de cada paciente es la suma total obtenida según la escala:

Clase 1: corresponde a un grupo con mínimo riesgo de complicaciones y definida por:

a) pacientes ≤ 50 años y sin las enfermedades reseñadas en la escala, y b) ausencia de los siguientes hallazgos físicos: estado mental alterado; pulso ≥ 125/min; frecuencia respiratoria ≥ 30/min; presión arterial (PA) sistólica < 90 mmHg; temperatura (Tª) < 35° C o ≥ 40° C.

Clase 2: si la puntuación es < 70.

Clase 3: puntuación entre 71-90.

Clase 4: puntuación entre 91-130.

Clase 5: puntuación > 130.

ANEXO II. CALENDARIO DE VISITAS DE PACIENTES CON NAC AMBULATORIA

**Visita 1 (Día 1)**	**Visita 2 (Día 2)**	**Visita 3 (telefónica) (Día 7)**	**Visita 4 (Día 30)**
Criterios de inclusión			
Anamnesis	Reevaluación médica	Evolución clínica	Evolución clínica
Exploración física	Reevaluación médica		Evolución clínica
Temperatura	Temperatura		
Tensión arterial	Tensión arterial[d]		
Bioquímica urgente	Bioquímica completa	Ver resultados bioquímica	[e]Bioquímica
Hemograma urgente	Hemograma	Ver resultados hemograma	[e]Hemograma
Gasometría arterial[a]	Gasometría arterial[a]		
Hemocultivos[b]	Hemocultivos[b]		
Puntuación Fine[c]			
	Extracción de suero archivo		Extracción de suero archivo
	Cultivo esputo		
	Antígenos en orina		
	Solicitud RX tórax para visita 4		Valoración RX tórax
Tratamiento<br>Oral<br>Parenteral	Tratamiento<br>Oral<br>Parenteral	Respuesta al tratamiento	Respuesta al tratamiento
Citación visita 2	Citación visita 3	Citación visita 4	Informe alta
			Alta

[a]Sólo en los siguientes casos: taquipnea, disnea, hipotensión arterial, mal estado general del paciente.
[b]Sólo en los siguientes casos: hipotensión arterial, tiritona en el momento de la evaluación, hipotermia<35ºC ó hipertermia>=40ºC.
[c]Ver Anexo 1
[d]Sólo en los siguientes casos: taquipnea, disnea, hipotensión arterial, mal estado general del paciente.
[e]Sólo si leucocitosis>15.000, anemia, elevación de transaminasas, alteraciones hidroelectrolíticas en analítica previa.
