# Supplementary material for: Procalcitonin for selecting the antibiotic regimen in outpatients with low-risk community-acquired pneumonia using a rapid point-of-care testing: A single-arm clinical trial
Source: PLoS One. 2017 Apr 20;12(4):e0175634. doi: 10.1371/journal.pone.0175634 (PMC5398537; doi:10.1371/journal.pone.0175634)
Supplement: S2 File — (PDF) [file pone.0175634.s003.pdf]

Title: Clinical pathway based on the levels of procalcitonin for the management of outpatients with community-acquired pneumonia

Key words: Clinical pathway, community-acquired pneumonia, outpatient treatment, evaluation, indicators.

Principal investigator: Mar Masiá Canuto

Institution: Hospital General Universitario de Elche

Address: Camí de la Almazara, 11

C. POSTAL: 03203

Location: Elche

Province: Alicante

Phone: 96 6616754

1. SUMMARY:

Aim: (1) To systematize and order the management of community- acquired pneumonia (CAP) that does not require hospital admission through the development of a clinical pathway and to define indicators for evaluation; (2) To assess the role of the procalcitonin measured with a rapid point-of-care test to select the composition of antibiotic regimen in outpatients with CAP; (3) To assess the efficacy and safety of the intervention and compare both with a historical control group; (4) To determine the mortality in the short-term (30 days) and long-term (3 years or longer) and the frequency of recurrences.

Design: Population-based prospective study.

Scope of the study: Área sanitaria del Baix Vinalopó.

Study subjects: Patients with community-acquired pneumonia not requiring hospitalization (Pneumonia Severity Index [PSI] scores I and II).

Instrumentalization: All patients: anamnesis, physical examination, chest XR, collection of blood and sputum samples for analysis, which will include serum procalcitonin measurement and microbiological studies, and dispensing and instructions on antibiotic treatment to be followed.

Measurements: Identification of the main pathogens involved in pneumonia through the culture of sputum and determination of antigens in urine. Evaluation of response to treatment, incidence of complications, adverse events, recurrences, mortality, and compliance with the clinical pathway. Estimation of the impact on the effectiveness and efficiency.

2. BACKGROUND AND JUSTIFICATION OF THE STUDY:

Variability in clinical practice or disparity of criteria to attend the same medical process is a common fact that poses a threat on the effectiveness of health services, the efficiency of its management and equity in its provision (1). Clinical pathways are instruments that allow planning and coordinating the health care of a group of patients with a pathology well defined and a predictable clinical course, through the definition of the set of activities and interventions in health care by doctors, nurses and other health professionals, arranging them sequentially in time (2). Thus, they seek to contribute to making a more proper use, rational and coordinated from existing health resources to achieve maximum efficiency and quality in the health care process.

Community-acquired pneumonia (CAP) is a serious and common process. In the health area of the Baix Vinalopó, incidence rate is 12 cases per 10,000 per year, which is a major cause of morbidity, mortality, and a considerable financial burden. The available evidence suggests that there is significant variability in the clinical management of the CAP (3, 4), and there are aspects in the care of this pathology which are subsidiary to be improved (5, 6). The implementation of a clinical pathway to the patients with CAP could help to optimize the management of this disease. In fact, different interventions aimed at improving care for these patients that also contribute to improve their clinical course, such as the assessment of the severity of pneumonia at the time of diagnosis, the time to receive the first doses of the antibiotic or prescribing appropriate antibiotic (7).

In recent years, the impact of the implementation of a clinical pathway patients with CAP has been evaluated, primarily those who require hospital admission and an improvement in the quality of care for patients was found (8-10). However, there is little experience on the usefulness of the implementation of a clinical pathway in patients with CAP that do not require hospital admission. From a theoretical point of view, the systematization of the care process of the CAP that does not require hospital admission through the introduction of a clinical pathway would allow saving admissions through the identification of low-risk patients according to the severity score PORT index (11), saving unnecessary diagnostic tests, improving the clinical course of patients with the introduction of measures such as early antibiotic treatment and increased efficiency in the care of this disease (12).

Procalcitonin (PCT) is a precursor of the hormone calcitonin and its levels have been found elevated during bacterial infection [13]. In patients with NAC, PCT levels have been associated with the microbial etiology; typical bacterial NAC, particularly pneumococcal infection, has been associated with significantly higher levels of PCT that infection by atypical pathogens (*Mycoplasma, Legionella and Chlamydophila Coxiella*) or viral pneumonia [14-18]. The PCT has been evaluated extensively in the treatment of lower respiratory infections, especially to guide the initiation and discontinuation of antibiotic treatment [18-21].

3. REFERENCES

1. Esteve M, L Marbá, Zaldivar C, Verdaguer A, Serra-Prat M, Berenguer J. Evaluación of the start-up of 54 clinical paths in the Hospital de Mataró. Rev quality of care 2001; 722-16: 28.
2. Carrasco G, Ferrer J. Clinical pathways based on the evidenciaz as a strategy for improving the quality: methodology, advantages, and limitations. RTVE quality health care 2001; 16: 199-207.
3. Laurichesse H, Robin Gerbaud L, F, Gourdon, Pochet P, Beytout J, King M. Empirical therapy for nonhospitalized patients with community-acquired pneumonia. Study Group of General Practitioners. EUR Respir J 1998; 11: 73-8.
4. Woodhead M, Leophonte P, Gialdroni Grassi G, Huchon GJ, Manresa F, Schaberg T. Use of investigations in lower respiratory tract infection in the community: a European survey. EUR Respir J 1996; 9: 1596-600.
5. Minogue MF, Coley CM, Fine MJ, Marrie TJ, Kapoor WN, Singer of. Patients hospitalized after initial outpatient treatment for community-acquired pneumonia. Ann Emerg Med 1998; 31: 376-80.
6. Metlay JP, Kapoor WN, Fine MJ. Does this patient have community-acquired pneumonia? Diagnosing pneumonia by history and physical examination. JAMA 1997; 278: 1440-5.
7. Nathwani D, F Williams, j. Winter, Winter J, Ogston S, Davey P. Use of indicators to evaluate the quality of community-acquired pneumonia management. Clin Infect Dis 2002; 34: 318-23
8. Meehan TP, Weingarten SR, Holmboe is, Mathur D, Wang Petrillo MK, your GS, Fine JM. A statewide initiative to improve the care of hospitalized pneumonia patients: The Connecticut Pneumonia Pathway Project. Am J Med 2001; 111: 203-10.
9. Marrie TJ, Lau CY, Wheeler SL, Wong CJ, Vandervoort MK, Feagan BG. A controlled trial of a critical pathway for treatment of community-acquired pneumonia. CAPITAL Study Investigators. Community-Acquired Pneumonia Intervention Trial Assessing Levofloxacin. JAMA 2000; 283: 749-55.
10. Benenson R, Magalski A, Cavanaugh S, Williams E. Effects of a pneumonia clinical pathway on time to antibiotic treatment, length of stay, and mortality. ACAD Emerg Med 1999; 6: 1243-8.
11. Fine MJ, Aublé TE, Yealy DM, Hanusa BH, Weissfeld LA, Singer DE, Coley CM, Marrie TJ, Kapoor WN. A prediction rule to identify low-risk patients with community-acquired pneumonia. N Engl J Med 1997; 336:243-50.
12. Nathwani D, Rubinstein E, Barlow G, Davey P. Do guidelines for community-acquired pneumonia improve the cost-effectiveness of hospital care? Clin Infect Dis 2001; 32: 728-41.
13. Becker KL, Nylen is, White JC, Muller B, Snider RH Jr. Procalcitonin and the calcitonin family of peptides in inflammation, infection, sepsis and gene: a journey from calcitonin back to its precursors. J Clin Endocrinol Metab. 2004; 89:1512-25

14. Kruger S, Ewig S, Papassotiriou J, Kunde J, Marre R, von Baum H, et a. Inflammatory parameters predict etiologic patterns but do not allow for single

prediction of etiology in patients with CAP - Results from the German competence network CAPNETZ. *Respir Res. 2009;* 10: 65.

15. F. Müller, Christ-Crain M, Bregenzer T, Krause M, Zimmerli W, Mueller B, et a. Procalcitonin levels predict bacteremia in patients with community-acquired pneumonia: a prospective cohort trial. *Chest.* 2010; 138:121-9.

16. Prat C, Dominguez Andreo F, White S, Pallares to knife F, et al the. Procalcitonin and neopterin with aetiology and severity of pneumonia. correlation *J Infect.* 2006; 52:169-77

17. Farmhouse M, Gutiérrez F, C Shum, Padilla S, Navarro JC, flowers E, et to the. Usefulness of procalcitonin levels in community-acquired pneumonia according to the patients outcome research team pneumonia severity index. Chest. 2005; 128:2223-9

18. Branche AR, Walsh EE, Vargas R, Hulbert B, Formica MA, Baran a., et to the. Serum Procalcitonin Measurement and Viral Testing to Guide Antibiotic Use for Respiratory Infections in Hospitalized Adults: A Randomized Controlled Trial. J Infect Dis. 2015 [Epub ahead of print]

19. Christ-Crain's M, Jaccard-Stolz D, Bingisser R, Gencay MM, Huber PR, Tamm M, et to the. Effect of procalcitonin-guided treatment on antibiotic use and outcome in lower respiratory tract infections: cluster-randomised, single-blinded intervention trial. Lancet. 2004; 363:600-7.

20. Kristoffersen KB , Søgaard OS, Wejse C, Black FT, Greve T, Tarp B, et to the. Antibiotic treatment interruption of suspected lower respiratory tract infections based on a single procalcitonin measurement at hospital admission - a randomized trial. Clin Microbiol Infect. 2009; 15:481-7

21. Schuetz P, Muller B, Christ-Crain M, Stolz D, Tamm M, Bouadma L, et to the. Procalcitonin to initiate or discontinue antibiotics in acute respiratory tract infections. Cochrane Database Syst Rev. 2012; 9:CD007498

4. HYPOTHESIS AND SPECIFIC OBJECTIVES

Hypothesis:
The systematization of the care process of community-acquired pneumonia that does not require hospital admission through the development and implementation of a clinical pathway would make it possible to improve the effectiveness and efficiency in the care of this pathology. The use of procalcitonin to guide the choice of the antibiotic treatment is a safe strategy that will allow saving the use of fluoroquinolones

Specific objectives:

1. To systematize and order the care process of pneumonia acquired in the community that does not require hospital admission through the development of a clinical pathway and to define indicators for evaluation.
2. To evaluate the role of procalcitonin measurement with a rapid test to select the composition of antibiotic treatment in patients with community pneumonia not requiring hospitalization.
3. To assess the efficacy and safety of the intervention and to compare them with a historical control group
4. To determine the short-term (30 days) and long-term (at least 3 years) mortality and the frequency of recurrences.

Outcome Measures:

Primary Outcome Measures:

- Clinical cure (Improvement or lack of progression of baseline radiographic findings at the end of therapy and resolution of signs, including chest X-Ray, and symptoms of pneumonia on day 30)

Secondary Outcome Measures:

- Number of participants with treatment-related adverse events
- Mortality (30-day and during the following 3 years or longer)
- Recurrences (new episodes of community-acquired pneumonia occurring after clinical cure of the initial episode through study completion, an average of 3 years)

5. METHODS

Study subjects: All patients visited in the hospital for community-acquired pneumonia not requiring hospitalization, i.e. belonging to PSI stages I and II.

Design: Population-based prospective study.

Variables: To evaluate the clinical path:

Collection and analysis of data: - A written protocol will be designed for collection of data that will include all patients' visits to the hospital. Data relating to the identification of patients, clinical features, procedures performed, treatment provided and time elapsed since the arrival to the hospital and administration of the first dose and development of complications will be collected.

-Analysis of the data: response to therapy, adverse events development, recurrences and mortality frequency will be collected. Clinical pathway indicators will be determined through the calculation of proportions, as described below.

Difficulties and limitations of the study:

- Appropriate coordination between the Emergency Service and the Infectious Disease Unit to ensure good adhesion to the Protocol.
- Insufficient recruitment of patients in the period of 3 months of the pilot study that allows the proper settings for the implementation of the final pathway.
- Difficulty in the calculation of the indicators of efficiency because of the retrospective data use for the comparison of pre-and post-implementation periods of the clinical pathway.

6. DURATION AND PLANNED PHASES:

1. Development of the clinical pathway and evaluation indicators
2. 3-month pilot study
3. Elaboration of the final clinical pathway
4. Implementation of the clinical pathway in the hospital
5. Quarterly revaluation
6. Assessment of impact indicators 1 year after the beginning of the pilot study

INCLUSION criteria : Patients must meet the following 3 criteria:

- ❑ Acute presentation of fever or any of the following signs/symptoms: change in the pattern of cough with or without expectoration, pleuritic chest pain, dyspnea, pathological pulmonary auscultation

  +

- ❑ Opacity in Xray compatible with the presence of pneumonia

  +

- ❑ PSI ≤ 70 points

EXCLUSION CRITERIA:

1. < 65 years age
2. Comorbidity
3. Pleural effusion
4. Bilateral involvement
5. Leukocytosis > 20,000
6. Risk factors for aspiration
7. Previous failure or macrolide or quinolone allergy
8. Need for oxygen therapy

SCHEDULE OF VISITS:

- There will be a total of 4 evaluations (1, 2, 3 and 4 visits), one of them (visit 3) by telephone contact.

The calendar of visits of patients is given in annex III.

PROCEDURES:

1. Anamnesis and physical examination: visit 1
2. Taking of vital signs: tours 1 and 2.
3. Collecting sputum culture: visit 2
4. Collection of urine for determination of antigens: visit 2
5. Extraction of blood for analysis: visits 1 and 2
6. Extraction of serum to freeze: visits 2 and 4
7. Realization of RX thorax: tours 1 and 4.

8. Only when the conditions are fulfilled detailed, extraction of blood cultures or blood gas analysis: 1 or 2 visits
9. Administration of the first antibiotic dose and instructions for the following doses: 1 to 2 visits.
10. Discharge report: visit 4.

INDICATORS FOR THE EVALUATION OF THE CLINICAL PATHWAY:

a) Indicators of the degree of compliance with the clinical pathway:

1. Number of patients who completed the clinical pathway x 100 / number of patients who are treated for pneumonia that does not require income. Standard: > 90%.

2. Average time elapsed since the arrival to the hospital until the administration of the first dose of the antibiotic. Standard: < 4 hours

(b) Indicator of adverse effects:

Number of outpatients with CAP who have any adverse event (need for hospital admission for clinical worsening, toxicity by antibiotics, development of empyema...) x 100 / number of outpatients with CAP following the clinical pathway. Standard: ≤ 5%.

Number of patients who die/number of patients included in the pathway

(c) Satisfaction indicator:

Number of outpatients with CAP who follow the pathway and answer with 8 or more on the question of care received in the survey x 100 / number of outpatients with CAP who follow the way and answered to the satisfaction survey. Standard: ≥ 98%.

(d) Economic evaluation indicator:

Number of outpatients with CAP who follow the pathway with adequate cost x 100 / number of outpatients with CAP who follow the pathway. Standard: > 90%.

INDICATORS OF THE IMPACT OF THE CLINICAL PATHWAY:

(a) Effectiveness:

-Proportion of patients with clinical cure without deviations from the protocol of the clinical pathway
-Decrease in the use of broad-spectrum antibiotic therapy
-Average time from arrival to the hospital until the administration of the first dose of the antibiotic. Standard: < 4 hours
-Proportion of patients who do not require change in the prescribed treatment

(b) Efficiency:

-Broad spectrum antibiotics consumption savings
-Saving the need for visits to the hospital.

DESCRIPTION OF THE CARE PROCESS:

VISIT 1 (day 1)
Area: Emergency department or Infectious Diseases Unit

(**b) Inclusion criteria:**

- ❑ Acute presentation of at least 1 of the following signs/symptoms: fever, change in the pattern of cough with or without coughing, dyspnea, pleuritic chest pain, pathological pulmonary auscultation

+

- ❑ Opacity in RX's compatible with the presence of pneumonia chest

+

- ❑ Fine ≤ 70 points

b) Medical evaluation

1. Anamnesis.
2. Physical examination.

(c) Findings and tests carried out by nurses:

1. Vital signs: blood pressure and temperature
2. Blood for urgent analytic (Biochemistry and blood cell count)
3. Extraction of arterial blood gases in the following cases: tachypnea, dyspnea, hypotension and poor general condition of the patient.
4. Extraction of 2 blood cultures in the following cases: hypotension, chills at the time of the evaluation, hypothermia < 35 ºc or hyperthermia > = 40 ° C.

(d) Criteria for outpatient management:

Score according to PSI scale (see annex 1 to the end) less than or equal to 70 points, or stages I and II.

(e) Treatment assignation:

- ❑ Procalcitonin< 0.50 ug/l: azithromycin, 500 mg/day P.O
- ❑ Procalcitonin ≥ 0.50 ug/L : levofloxacin 500 mg/day P.O

(f) Citation for reassessment:

Refer the patient to infectious disease unit to be seen the next day (*visit 2*).

VISIT 2 (day 2)
Area: Infectious Diseases Unit

(a) Medical reassessment

(b) Determinations and tests carried out by nurses:

1. Measured temperature and, only in the following cases, blood pressure: low blood pressure the previous day, chills at the time of the evaluation, signs of hypoperfusion (important sweating, coldness), poor general condition.
2. Blood for general blood tests (complete Biochemistry and blood cell count)
3. Serum file extraction to freeze
4. Urine collection for determination of antigens of pneumococcus and *Legionella*
5. Collecting sputum for Gram and culture
6. Extraction of arterial blood gases in the following cases: tachypnea, dyspnea, hypotension and poor general condition of the patient.
7. Extraction of 2 blood cultures in the following cases: hypotension, chills at the time of the evaluation, hypothermia < 35 ºc or hyperthermia > = 40 ° C.

(c) Treatment assignation:

❑ Procalcitonin < 0.50 ug/l: azithromycin, 500 mg/day P.O 5 days

❑ Procalcitonin ≥ 0.50 ug/L: levofloxacin 500 mg/day P.O 10 days

(d) Citation for reassessment:
Schedule for *visit 3*, which will take place by telephone contact, within 1 week, or earlier if it is indicated by the patient's clinical status.

VISIT 3 (day 7): Phone visit

(a) Medical evaluation

1. Assessment of clinical progression and response to treatment
2. Assessment of the results of the tests performed in the visit 2

(b) Appointment for reassessment:

Appointment for *visit 4* at 3 weeks. Chest X ray will be performed in visit 4 and blood tests (serum sample for freezing and general analyses if indicated).

VISIT 4 (day 30)
Area: Infectious Diseases Unit

Medical evaluation:

1. Assessment of clinical progression and response to treatment
2. Assessment of new the chest X ray

Measurements and tests carried out by nurses:

1. Extraction of a serum sample to be kept frozen.
2. General blood tests, only in cases in which there are significant pathologic findings in the prior analytics.
3. Discharge from the Infectious Diseases Unit, unless blood tests were drawn. In this case, informed about the result to the patient by telephone within days.
4. Elaboration of the discharge report.

Annex 1. Pneumonia severity index score

Annex II. Schedule of visits of patients with OUTPATIENT CAP

**Visit 1 (day 1)**	**Visit 2 (day 2)**	**Visit 3 (phone) (day 7)**	**Visit 4 (30 day)**
Inclusion criteria			
Anamnesis	Medical reassessment	Clinical evolution	Clinical evolution
Physical examination	Medical reassessment		Clinical evolution
Temperature	Temperature		
Blood pressure	[D] blood pressure		
Urgent biochemistry	Comprehensive biochemistry	See biochemical results	[e] Biochemistry
Urgent blood count	Blood count	See complete blood count results	[e] Blood count
Arterial blood gas[to]	Arterial blood gas[to]		
Blood cultures[b]	Blood cultures[b]		
Score Fine[c]			
	Serum sample extraction		Serum sample extraction
	Sputum culture		
	Antigens in urine		
	Request RX thorax for visit 4		Evaluation chest X ray
Treatment<br>Oral<br>Parenteral	Treatment<br>Oral<br>Parenteral	Response to treatment	Response to treatment
Citation visit 2	Citation visit 3	Citation visit 4	Discharge report
			Discharge

[to] Only in the following cases: tachypnea, dyspnea, hypotension and poor general condition of the patient.

[b] Only in the following cases: hypotension, tiritona at the time of the evaluation, hypothermia < 35 ºc or hyperthermia > = 40 ° c

[c] See annex 1

d Only in the following cases: tachypnea, dyspnea, hypotension and poor general condition of the patient.

[e] Only if Leukocytosis > 15,000, anemia, elevated transaminases, alterations imbalance in previous analytical.
